# Development of a 3-Dimensional Model to Study Right Heart Dysfunction in Pulmonary Arterial Hypertension: First Observations

**DOI:** 10.3390/cells10123595

**Published:** 2021-12-20

**Authors:** Aida Llucià-Valldeperas, Rowan Smal, Fjodor T. Bekedam, Margaux Cé, Xiaoke Pan, Xue D. Manz, Paul J. M. Wijnker, Anton Vonk-Noordegraaf, Harm J. Bogaard, Marie-Jose Goumans, Frances S. de Man

**Affiliations:** 1 PHEniX Laboratory, Department of Pulmonary Medicine, Amsterdam UMC, Vrije Universiteit Amsterdam, Amsterdam Cardiovascular Sciences, 1081 HZ Amsterdam, The Netherlands; a.lluciavalldeperas@amsterdamumc.nl (A.L.-V.); r.smal@amsterdamumc.nl (R.S.); f.t.bekedam@amsterdamumc.nl (F.T.B.); m.j.a.ce@amsterdamumc.nl (M.C.); x.pan@amsterdamumc.nl (X.P.); x.manz@amsterdamumc.nl (X.D.M.); a.vonk@amsterdamumc.nl (A.V.-N.); hj.bogaard@amsterdamumc.nl (H.J.B.); 2 Department of Physiology, Amsterdam UMC, Vrije Universiteit Amsterdam, Amsterdam Cardiovascular Sciences, 1081 HZ Amsterdam, The Netherlands; p.wijnker@amsterdamumc.nl; 3 Department of Cell and Chemical Biology, Leiden UMC, 2300 RC Leiden, The Netherlands; M.J.T.H.Goumans@lumc.nl

**Keywords:** pulmonary arterial hypertension, right heart dysfunction, induced pluripotent stem cells, engineered heart tissue

## Abstract

Pulmonary arterial hypertension (PAH) patients eventually die of right heart failure (RHF). Currently, there is no suitable pre-clinical model to study PAH. Therefore, we aim to develop a right heart dysfunction (RHD) model using the 3-dimensional engineered heart tissue (EHT) approach and cardiomyocytes derived from patient-induced pluripotent stem cells (iPSCs) to unravel the mechanisms that determine the fate of a pressure-overloaded right ventricle. iPSCs from PAH and healthy control subjects were differentiated into cardiomyocytes (iPSC-CMs), incorporated into the EHT, and maintained for 28 days. In comparison with control iPSC-CMs, PAH-derived iPSC-CMs exhibited decreased beating frequency and increased contraction and relaxation times. iPSC-CM alignment within the EHT was observed. PAH-derived EHTs exhibited higher force, and contraction and relaxation times compared with control EHTs. Increased afterload was induced using 2× stiffer posts from day 0. Due to high variability, there were no functional differences between normal and stiffer EHTs, and no differences in the hypertrophic gene expression. In conclusion, under baseline spontaneous conditions, PAH-derived iPSC-CMs and EHTs show prolonged contraction compared with controls, as observed clinically in PAH patients. Further optimization of the hypertrophic model and profound characterization may provide a platform for disease modelling and drug screening.

## 1. Introduction

The heart is a marvelous and complex organ that scientists repeatedly try to replicate in laboratories, with more or less success, in order to study cardiovascular diseases, which are still the leading cause of death in developed countries. Within these fatal diseases, we also encounter pulmonary arterial hypertension (PAH), a rare disease that originates in the lungs but has devastating consequences for the heart. PAH is characterized by pulmonary vascular remodeling that causes up to a fourfold rise in pulmonary artery pressure. The right ventricle adapts to the increasing vascular load and maintains blood flow by enhancing contractility. However, the right ventricle cannot cope indefinitely with the increased afterload, and eventually, patients die of right heart failure (RHF) [1]. Unfortunately, there is no specific treatment for RHF and current options aim to reduce the pulmonary vascular resistance or to use drugs tested for left heart failure. The development of RHF-specific treatment has been hampered by limited availability and suitability of human samples and representative animal models. First, human right ventricle biopsies from PAH patients are scarce and restricted to the end-stage status. From end-stage RHF tissue, it is impossible to obtain insights into the role of, e.g., genetics or sex on the *response* of the right ventricle to pressure overload [2,3]. Only information on the *effect* of prolonged pressure overload on the right ventricle can be obtained. Second, animal models do not completely reproduce the pathophysiology of the right ventricle in PAH. Hence, there is a need for the development of a novel and clinically relevant preclinical model to investigate RHF and right heart dysfunction (RHD).

Human-induced pluripotent stem cells (iPSCs) are a promising source for the production of patient-specific cells, such as cardiomyocytes. Few publications succeed in generating iPSCs from PAH patients [4,5]. Since the discovery of iPSCs in 2006 [6], several approaches have been established to efficiently derive cardiomyocytes from human iPSCs (iPSC-CMs) [7]. iPSC-CMs cultured on monolayers were a huge step forward in the field and gave important information on cardiomyocyte contractile performance. However, because of the inherent limitations of a 2-dimensional (2D) approach, researchers have aimed to develop a 3-dimensional (3D) configuration that better resembles cardiac tissue physiology. Regarding 3D strategies, cardiac tissue engineering is a continuously growing field in biomedicine and has already provided advanced in vitro models for drug testing and disease modeling. The 3D engineered heart tissue (EHT) approach emerged as the optimal method for miniaturization, multi-well testing, and automated evaluation [8]. EHTs recapitulate tissue organization in vitro to study cell–cell interactions and cardiac function under healthy and pathological conditions [9]. Therefore, EHTs, together with the Langendorff setup and the zebrafish model, can fill the translational gap between isolated cardiomyocytes and animal models [10].

In this study, we aimed to engineer a model to study PAH-induced RHD using iPSC-CMs from PAH patients and to simultaneously investigate the baseline contractile features of iPSC-CMs in 2D and 3D models.

## 2. Materials and Methods

### 2.1. Generation of Human iPSCs 

Late-outgrowth endothelial colony-forming cells (ECFCs) were isolated and cultured from peripheral blood from one female healthy subject (28 years old at the time of blood collection) and one female PAH patient (27 years old at the time of blood collection, 20 mm Hg for mean pulmonary arterial pressure, and 52% right ventricular ejection fraction), as previously described [11]. A heterozygous mutation on the Bone Morphogenetic Protein 2 (BMPR2) gene (missense mutation c.1454A>G) was present in the PAH patient and absent in the healthy subject. In brief, the mononuclear cell fraction was isolated from peripheral blood using Ficoll density centrifugation. Next, cells were resuspended in EGM-2 medium (EBM-2 with single quotes growth factor-kit, Lonza, Basel, Switzerland) supplemented with 10% human platelet lysate and seeded on type I collagen-matrix (rat tail collagen, BD, Franklin Lakes, NJ, USA). Culture plates were kept at 37 °C and 5% CO_2_. Non-adherent cells were removed after 3 days, and the culture medium was refreshed every other day. ECFC colonies appeared after 7–28 days and were successively expanded.

Subsequently, three iPSCs clones were generated from each ECFCs population using a lentiviral approach containing the four Yamanaka factors by the LUMC hiPSC Hotel [12]. In short, cells were transduced and seeded onto a fresh layer of irradiated mouse embryonic fibroblasts. Visible iPSCs colonies appeared around week 3, which were manually transferred into a Vitronectin (StemCell Technologies, Vancouver, Canada)-coated 6-well plate in TeSR-E8 medium (StemCell Technologies) at 37 °C and 5% CO_2_ for further expansion. The iPSCs karyotype was routinely checked to ensure no chromosomal aberrations were present in the cultures. The presence or absence of the BMPR2 mutation was confirmed in all iPSCs clones by Sanger sequencing. All iPSCs clones were characterized for their pluripotency capacity at both the gene and protein levels, tested by culturing them at a low cell density under spontaneous differentiation basal conditions (DMEM/F12 medium supplemented with 20% Fetal Bovine Serum, Thermofisher Scientific, Waltham, MA, USA) for 3 weeks and by analyzing the resulting lineages through immunostaining, as explained later. All three iPSCs clones/subject were grouped as a control or PAH for the analyses.

Patient inclusion and blood collection were approved by the Institutional Ethical Review Board of the VU Medical Center (study number METC VUmc 2015.220/NL53211.029.15) and no signed informed consent was required for a single blood draw before 7th July 2017. This investigation conformed to the principles outlined by the Declaration of Helsinki.

### 2.2. Cardiac Differentiation of iPSCs

**Figure 1:**
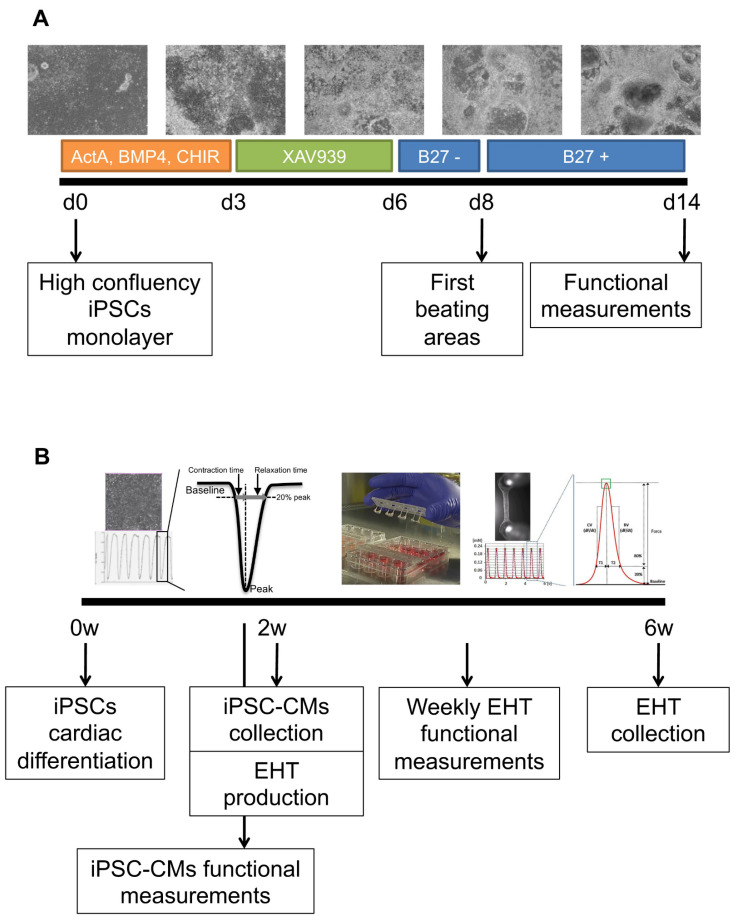
Schematic of the protocol for the differentiation of cardiomyocytes from human iPSCs and complete EHT experimentation. (**A**) Cardiac differentiation consisted of a first step with 20 ng/mL of BMP4, 20 ng/mL of activinA, and 1.5 µM of CHIR99021 for mesoderm induction, followed by a second step with 5 µM of XAV939 for WNT signal inhibition and cardiac specification, and finally, insulin was added from day 8, after the first beating areas were observed. (**B**) The experimental design started with iPSCs cardio-differentiation and baseline functional measurements before the iPSC-CMs were collected and used for EHT production; later, EHT contractility was measured twice a week for 28 days until the end of the experiment.

Human iPSCs were differentiated into cardiomyocytes using a new optimized protocol derived from previous publications [13,14,15] (Figure 1A). In short, a confluent monolayer of human iPSCs was differentiated by adding RPMI media (Thermofisher Scientific) supplemented with B27 minus insulin (Thermofisher Scientific), 50 μg/mL of ascorbic acid (Thermofisher Scientific), 20 ng/mL of BMP4 (R&D Systems, Minneapolis, MN, USA), 20 ng/mL of activinA (StemCell Technologies), and 1.5 μM of CHIR99021 (TOCRIS, Bristol, UK) for 3 days. Next, the medium was changed to an RPMI medium supplemented with B27 minus insulin, 50 μg/mL of ascorbic acid, and 5 μM of XAV939 (TOCRIS) for 3 more days; later, the cells were cultured with an RPMI medium supplemented with B27 minus insulin and 50 μg/mL of ascorbic acid for 2 days. From day 7, the first beating areas appeared and the medium was refreshed with an RPMI medium supplemented with B27 plus insulin (Thermofisher Scientific) and 50 μg/mL of ascorbic acid, with a media change every other day.

### 2.3. Generation of EHTs

EHTs were produced as previously explained [8,16], and the experimental design is described in Figure 1B. Briefly, iPSC-CMs at day 14 were mechanically selected from the differentiation plate and digested with 5 mg/mL of collagenase type 1 (Worthington Biochemical Corporation, Lakewood, NJ, USA) for 45 min at 37 °C under agitation and later with 2 mg/mL of collagenase type 2 (Worthington Biochemical Corporation) for 45 min at 37 °C. Dissociated iPSC-CMs were counted (~1–2 × 10^6^ cells) and combined with 5 mg/mL of bovine fibrinogen (Merck, Darmstadt, Germany), 100 μL/mL of Matrigel (BD), DMEM (2 × 1 g/L D-Glucose, Biochrom), and 0.25 U/mL of thrombin (Merck), pipetted into the 2% agarose (Thermofisher Scientific) molds previously solidified in a 24-well culture dish with the silicone posts racks. After 90 min at 37 °C and 7% CO_2_, 300 μL of the cell culture medium was added to easily remove the EHTs, and they were transferred into a new 24-well plate. EHTs were maintained at 37 °C in a 7% CO_2_ humidified cell culture incubator with media changes three times a week and functional measurements twice a week. The EHT medium consisted of DMEM (Biochrom, Cambridge, UK), 10% inactivated horse serum (Merck), 1% penicillin/streptomycin (Merck), 10 μg/mL of insulin (Merck), and 33 μg/mL of aprotinin (Merck).

### 2.4. Development and Characterization of the Stiffer Silicone Posts

In this study, two types of silicone posts were used for the generation of the EHTs: normal posts for physiological conditions and stiffer posts to mimic the increased afterload observed in the dysfunctional right ventricle. Silicone posts with normal stiffness were purchased from EHT technologies (DiNAQOR, Zurich, Switzerland). Silicone posts with increased stiffness were custom-made in our workplace, adapting the dimensions specified in a previous publication ([16], Online Figure 1A). Briefly, sylgard 184 silicone elastomer (Dow Corning, Midland, MI, USA) was used to produce the posts in custom-made Teflon casting molds. Each rack carried four pairs of silicone tubes instead of pillars, where the lower apertures of the tubes were closed by silicone discs to avoid the entrance of the medium and to ameliorate EHT adherence.

Normal and stiffer posts were elastic and obeyed Hooke’s law for springs. Hence, the resistance of the posts against the beating EHT could be calculated and expressed as a force or spring constant (N/m). In order to measure the spring constant of the silicone posts, single posts were cut from the silicone rack and mounted onto a solid platform. This platform was placed in a setup (ASI 802D, Aurora Scientific Inc., Aurora, ON, Canada) that was designed to measure force and length changes in single muscle fibers. A length motor (ASI 315C-I, Aurora Scientific Inc.,) and a force transducer (ASI 403A, Aurora Scientific Inc) were used to measure force in relation to the deflection of the silicone post. The spring constant was thereby determined for the physiological range that EHTs reached during contractions in between a set of silicone posts. The stiffer posts had two times more resistance than normal posts, and increased afterload was imposed on the EHTs from day 0 using these stiffer posts.

### 2.5. Immunofluorescent Staining

iPSCs and iPSC-CMs were fixed in 4% paraformaldehyde in phosphate-buffered saline (PBS, pH 7.4) for 20 min at room temperature. Samples were permeabilized, blocked, and incubated at room temperature for 1 h with primary antibodies against NANOG (diluted 1:100; Santa Cruz Biotechnologies, Dallas, TX, USA), SSEA-4 (diluted 1:30; Biolegend, Perkin Elmer, San Diego, CA, USA), OCT-3/4 (diluted 1:100; Santa Cruz Biotechnologies), β-tubulin-3 (TUBB3, diluted 1:4000; Biolegend), alpha fetoprotein (AFP, diluted 1:25; Quartett, Berlin, Germany), PECAM (diluted 1:50; Dako, Agilent, Santa Clara, CA, USA), cardiac troponin T (TNNT2, diluted 1:500; Abcam, Cambridge, UK), sarcomeric α-actinin (ACTN1, diluted 1:200; Merck), myocyte enhancer factor-2 (MEF2, diluted 1:200; Santa Cruz biotechnologies), or vimentin (diluted 1:500; Abcam). Samples were then incubated for 1 h at room temperature with secondary antibodies conjugated with Alexa-488, Alexa-555, or Alexa-647 (diluted 1:500; Abcam). The cells were counterstained with Hoechst 33342 nuclear dye (diluted 1:500; Santa Cruz Biotechnology) and actiStain phalloidin conjugated to Alexa-670 (diluted 1:200; Cytoskeleton Inc., Denver, CO, USA).

EHTs were fixed overnight at 4 °C using 4% paraformaldehyde in PBS, and whole-mount staining was performed. EHTs were removed from the silicone posts; permeabilized; blocked at room temperature overnight; and incubated at room temperature for 24 h with primary antibodies against TNNT2 (diluted 1:500; Abcam), cardiac troponin I (TNNI3, diluted 1:500; Abcam), ACTN1 (diluted 1:500; Merck), MEF2 (diluted 1:250; Santa Cruz biotechnologies), and vimentin (diluted 1:500; Abcam). Samples were then incubated for 6 h at room temperature with secondary antibodies conjugated with Alexa-488, Alexa-555, or Alexa-647 (diluted 1:500; Abcam). The cells were counterstained with Hoechst 33342 nuclear dye (diluted 1:500; Santa Cruz Biotechnology) and Alexa-647 conjugated wheat germ agglutinin (WGA, diluted 1:300; Thermofisher Scientific). 

Images were captured at 20×, 40×, and 60× magnifications under a laser confocal microscope (Nikon A1R, Nikon, Tokyo, Japan).

### 2.6. Quantitative Real-Time PCR Analysis

Total RNA was extracted from iPSCs and iPSC-CMs using Direct-zol RNA Miniprep kit with the DNase I step (Zymo Research, Irvine, CA, USA) following the manufacturer’s protocol. Total RNA was extracted from homogenized EHTs with the TissueLyser using the RNeasy kit (Qiagen, Hilden, Germany) with a preliminary chloroform step and, later, the DNase I step (Zymo Research) following manufacturer’s protocol. The concentration and purity of the RNA were measured on a Nanodrop One spectrophotometer (Thermofisher Scientific). Reverse transcription to cDNA was carried out using the iScript™ cDNA Synthesis Kit (Bio-Rad, Hercules, CA, USA). Quantitative real-time PCR amplifications were performed with 2 µL of cDNA in a final volume of 10 µL, containing 5 µL of Fast SYBR Green MasterMix (Thermofisher Scientific), 2 µL of RNase-free PCR-grade water (Thermofisher Scientific), and 1 µL of both forward and reverse primer solutions (sequences specified in Supplementary Table S1). Two housekeeping genes, glyceraldehyde-3-phosphate dehydrogenase (GAPDH), and ribosomal protein L27 (RPL27) or hypoxanthine phosphoribosyltransferase 1 (HPRT1), were used to ensure the validity and reproducibility of the results. Data were collected and analyzed in duplicate on the CFX384™ Real-Time System (C1000 Touch™ Thermal Cycler, Bio-Rad). Primers validated for pluripotency, spontaneous differentiation into the three germ layers, cardiac differentiation, and cardiac hypertrophy were used accordingly. The Livak method was used to quantify the relative (2^−ΔCT^) expression of each gene between groups.

### 2.7. Western Blot

iPSC-CM and EHT protein samples were collected in the lysis buffer (20 mM Tris-HCl, 150 mM NaCl, 100 mM KCl, 2 mM EDTA-NaOH, 5% Igepal, and 0.5% Triton X-100; pH 8.0) supplemented with phosphatase and protease inhibitor cocktail (Roche, Basel, Switzerland). Lysates were prepared with 1× NuPage LDS sample buffer (Thermofisher Scientific) and 50 µM DTT (Thermofisher Scientific). Protein samples were loaded on 4–12% NuPageTM Bis-Tris protein gel (Thermofisher Scientific) and electrophoresed at 200 V for ~1.5 h. Separated proteins were transferred to 0.45 µM Amersham Hybond ECL nitrocellulose membranes (Thermofisher Scientific) and blocked with 5% nonfat dry milk (Bio-rad) in Tris-buffered saline (pH 7.6) with 0.1% Tween (TBS-T) for 1 h at room temperature. Membranes were incubated overnight at 4 °C with gentle shaking in 5% nonfat dry milk with primary antibodies against TNNT2 (diluted 1:1000; Abcam), TNNI3 (diluted 1:1000; Abcam), ACTN1 (diluted 1:1000; Merck), MEF2 (diluted 1:200; Santa Cruz biotechnologies), ATPase sarcoplasmic/endoplasmic reticulum Ca^2+^ transporting 2 (ATP2A2, diluted 1:1000; Abcam), phospholamban (PLN, diluted 1:1000; Abcam), and vinculin (1:200, Merck). HRP-conjugated secondary antibodies (1:5000, Dako) were incubated in 5% nonfat dry milk for 1 h at room temperature. Bands were visualized with Amersham ECL Prime Blotting Detection Reagent (GE Healthcare Life Sciences, Eindhoven, The Netherlands), detected with the Amersham^TM^ Imager 600 (GE Healthcare Life Sciences), quantified with ImageJ (NIH, Bethesda, MD, USA), and normalized to vinculin expression.

### 2.8. Contractility Measurements

Cardiac contractility was assessed on spontaneously beating iPSC-CMs around day 14 before the cells were detached from the culture plate using the CytoCypher Multicell High Throughput System (CytoCypher BV, Amsterdam, The Netherlands). Briefly, the CytoCypher Multicell High Throughput System (CytoCypher BV) is a motorized stage microscope combined with a high-resolution camera and an objective that can move in x–y–z positions. iPSC-CMs contraction kinetics were measured at 37 °C and were based on pixel correlation changes relative to the reference frame taken at diastole at 250 Hz sampling frequency (IonOptix LLC, Westwood, MA, USA). Each area was measured for 10 s, in which ~10 contraction traces were recorded. We measured N ≥ 15 different areas per well and N ≥ 3 wells per differentiation batch. The CytoSolver Transient Analysis Tools package (CytoCypher BV) was used to yield averaged contractile and kinetic parameters from each area. For the purposes of this study, we analyzed the following key parameters of contraction: beating frequency (beats per minute (bpm)), contraction time (s), and relaxation time (s) at 80% of peak height. Areas were excluded from the analysis if they did not meet all inclusion criteria (R2 (peak fit) > 0.9, R2 (recovery fit) > 0.9, and R2 (single exponential fit) > 0.8). Moreover, the β-adrenergic response on iPSC-CMs was investigated throughout increasing isoprenaline (Merck) concentrations from 1 to 1000 nM every 10 min and the contractile measurements after 5 min at 37 °C using the CytoCypher Multicell High Throughput System (CytoCypher BV), as previously described.

Analyses of contractile force on EHTs were performed twice a week for 28 days by video optical recording on a setup available from EHT Technologies as previously described [8]. Briefly, the EHT setup consisted of a cell incubator unit controlling the level of CO_2_, humidity, and temperature and of a glass roof for monitoring using a camera positioned in the x–y–z directions. Video optical analysis was performed with a customized software package by Consulting Team Machine Vision based on automatic figure recognition of the contracting tissue at the top and bottom ends, post geometry, elastic modulus of the silicone posts, and post deflection. When stiffer silicone posts were used, the force was corrected by 2 because of the 2× higher resistance and according to the equation used to calculate the force [17]. We measured for 10 s N ≥ 8 EHTs per differentiation batch, condition (normal or stiff posts), and group (control or PAH) under spontaneous beating conditions. The contraction peaks were analyzed in terms of beating frequency (bpm), force (mN), contraction time (s), and relaxation time (s) at 80% of peak height.

### 2.9. Statistical Analysis

Statistical analyses were performed with R statistics package version 4.0.5 (R Foundation for Statistical Computing, Vienna, Austria) and R studio version 1.4.1106 (R-studio, Boston, MA, USA). Data are presented as mean + standard deviation (SD). Normality of the data was checked by a visual inspection using histograms and descriptive statistics. Functional differences between PAH and the control in 2D iPSC-CMs were analyzed by unpaired t-test with Bonferroni post hoc correction. Protein expression differences between PAH and the control in 3D EHTs were analyzed by unpaired t-test with Bonferroni post hoc correction. Functional differences between PAH and the control in 3D EHTs at 2–4 weeks were analyzed by paired t-test with Bonferroni post hoc correction. The effect of isoprenaline and stiff posts were assessed using a two-way repeated measures ANOVA test with Bonferroni post hoc correction. Results were considered statistically significant if the *p*-value was * *p* < 0.05, ** *p* < 0.01, and *** *p* < 0.001.

## 3. Results

### 3.1. Successful Production of iPSCs from PAH Patients and Control Subjects

**Figure 2:**
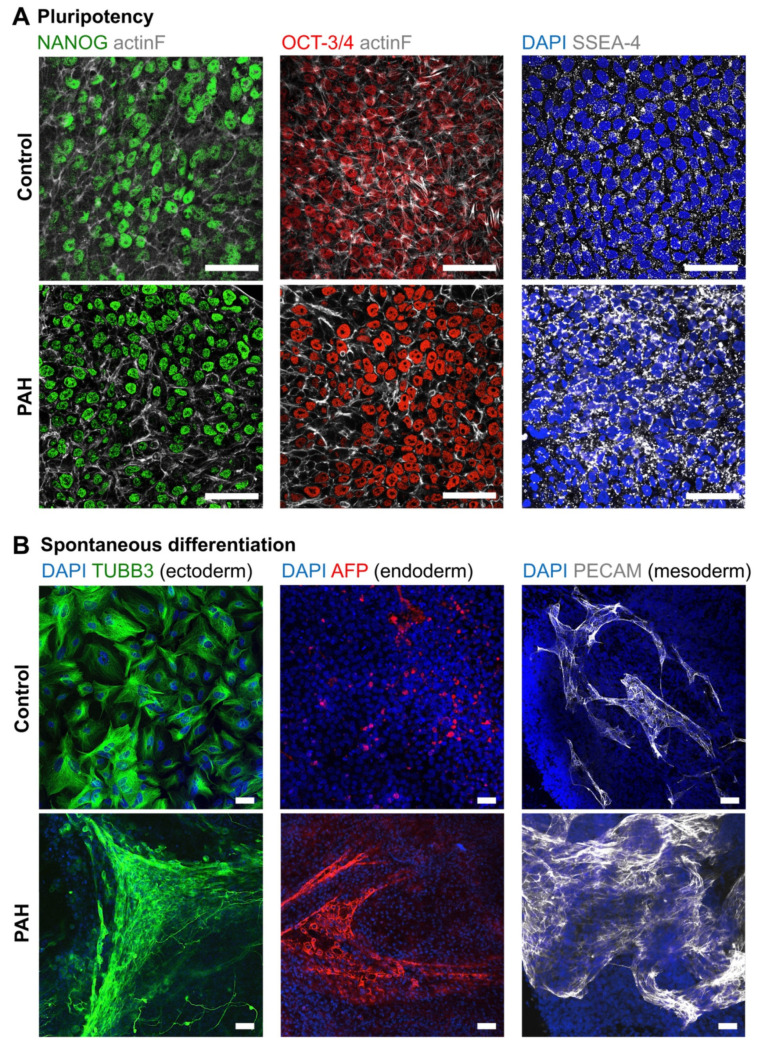
Pluripotency and spontaneous differentiation capacity of the control and PAH iPSCs. (**A**) Representative images confirming protein expression of pluripotency (NANOG, OCT-3/4, and SSEA-4) markers on the control and PAH iPSCs. (**B**) Representative images confirming protein expression of spontaneous differentiation markers (TUBB3, AFP, and PECAM) on the control and PAH iPSCs. Scale bars = 50 µm.

Three iPSCs clones reprogrammed from ECFCs isolated from the blood of one healthy subject and one PAH patient were characterized for their pluripotency capacity. Pluripotency markers (NANOG, OCT-3/4, and SSEA-4) or spontaneous differentiation markers of the three germ layers (TUBB3 for ectoderm, AFP for endoderm, and PECAM for mesoderm) were studied with iPSCs cultured with the TeSR-E8 maintenance medium or basal medium for 3 weeks, respectively (Figure 2A,B).

iPSCs cultured with TeSR-E8 maintenance medium expressed the pluripotent markers NANOG and OCT3/4 in the nuclei, and SSEA-4 in the plasma membrane (Figure 2A). Accordingly, when iPSCs were cultured with spontaneous differentiation basal medium, they differentiated into ectoderm, endoderm, and mesoderm, corroborated by positive TUBB3, AFP, and PECAM protein expressions, respectively (Figure 2B).

In summary, iPSCs spontaneously differentiated into the three germ layers when they were not cultured under optimal maintenance conditions.

### 3.2. iPSCs Differentiated into Cardiomyocytes

**Figure 3:**
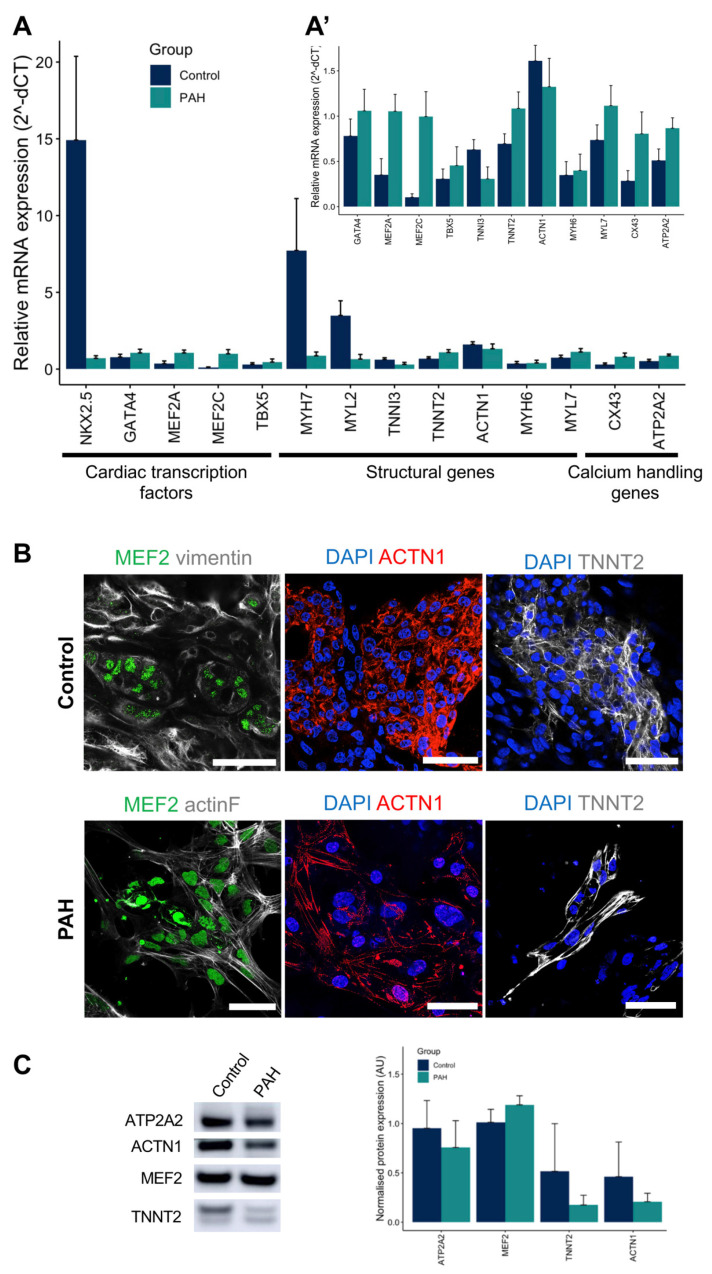
Control and PAH iPSC-CM characterization at both the gene and protein levels. (**A**) Gene expression of cardiac transcription factors (NKX2.5, TBX5, MEF2A, MEF2C, and GATA4), structural genes (ACTN1, MYH6, MYH7, MYL2, MYL7, TNNI3, and TNNT2), and calcium-handling genes (ATP2A2 and CX43) genes for the control and PAH iPSC-CMs (mean + SD, N = 6 for the control, and N = 6 for the PAH cardio-differentiation batches). (**A’**) Gene expression of previous genes except NKX2.5, MYH7, and MYH6 to appreciate smaller expressions in detail. (**B**) Representative images confirming nuclear expression of MEF2 (green), sarcomeric ACTN1 (red), and TNNT2 (grey) on the control and PAH iPSC-CMs. Scale bars = 50 µm. (**C**) Representative Western blot bands and protein quantification of ATP2A2, MEF2, TNNT2, and ACTN1 cardiac markers on the control and PAH iPSC-CMs (mean + SD, N = 5 control, and N = 4 PAH).

After iPSCs characterization, all control and PAH iPSC clones successfully differentiated into cardiomyocytes following our protocol and beating areas were visually observed between days 7 and 10 (Figure 1A, Supplementary Movie S1). iPSC-CMs expressed the main cardiac markers at both the gene and protein levels (Figure 3A–C). Control and PAH iPSC-CMs expressed early and late cardiac markers, such as cardiac transcription factors (NKX2.5, TBX5, MEF2A, MEF2C, and GATA4), structural genes (ACTN1, MYH6, MYH7, MYL2, MYL7, TNNI3, and TNNT2), and calcium-handling (ATP2A2 and CX43) genes (Figure 3A). Indeed, MEF2 and GATA4 proteins were observed in the nuclei, while TNNT2 and ACTN1 were located at the cytoplasm with a sarcomeric pattern on control and PAH iPSC-CMs (Figure 3B). The protein expression of ATP2A2, ACTN1, MEF2, and TNNT2 was also demonstrated and quantified for both control and PAH iPSC-CMs (Figure 3C).

Therefore, iPSC-CMs derived from control and PAH iPSC clones spontaneously beat and expressed main cardiac markers.

### 3.3. Control iPSC-CMs and PAH iPSC-CMs Reported Different Functional Properties

**Figure 4:**
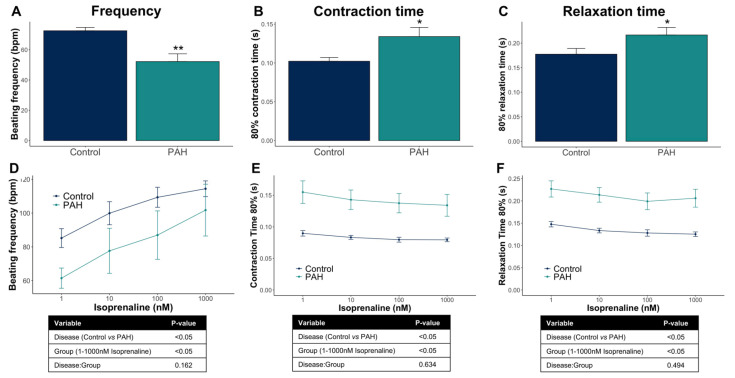
Functional analyses of the control and PAH iPSC-CM monolayers under spontaneous beating conditions. (**A**–**C**) Beating frequency (beats per minute, bpm), contraction time (s), and relaxation time (s) in the control and PAH iPSC-CMs (mean + SD, N = 46 control wells from seven different cardio-differentiations, and N = 16 PAH wells from five different cardio-differentiations). (**D**–**F**) Functional response of the control and PAH iPSC-CMs to increasing isoprenaline concentrations (nM) overtime (mean ± SD, N = 15 control, and N = 8 PAH). * *p* < 0.05, ** *p* < 0.01.

Fourteen days after the cardiac differentiation started, spontaneous cardiac contractility in iPSC-CM monolayers was evaluated based on pixel correlation changes relative to the reference frame using the CytoCypher Multicell High Throughput System (CytoCypher BV). Interestingly, PAH iPSC-CM monolayers showed prolonged contraction compared with control iPSC-CMs, evidenced by the decreased beating frequency and by the increased contraction and relaxation times (Figure 4A–C).

Furthermore, iPSC-CMs responded to β-adrenergic stimulation by increasing the beating frequency and by slightly decreasing contraction and relaxation times. Both the control and PAH iPSC-CMs have an analogous response to increasing concentrations of isoprenaline but different starting points because of the baseline contractile differences previously mentioned (Figure 4D–F).

Taken together, the control and PAH iPSC-CMs have different contractile kinetics but a similar response to isoprenaline treatment.

### 3.4. Optimization and Characterization of the 3D EHTs 

**Figure 5:**
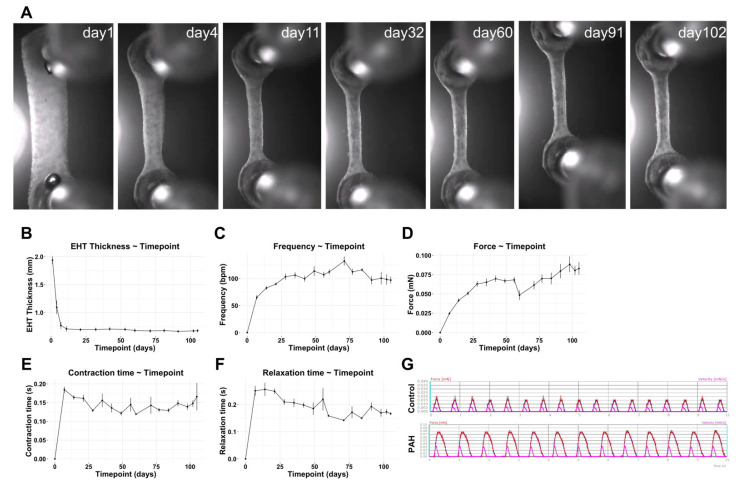
Long-term EHT experimentation and functional follow-up under spontaneous beating conditions. (**A**) Representative bright field images of 3D EHTs at different timepoints over 3 months. (**B**) Quantification of EHT thickness (mm) in the rectangular part of the 3D EHTs over time. (**C**–**F**) Beating frequency (bpm), force (mN), contraction time (s), and relaxation time (s) in 3D EHTs (mean ± SD, N = 8 PAH EHTs). (**G**) Representative twitch tracings for the control and PAH EHTs for 10 s (force (mN) in red and velocity (mN/s) in pink).

Human iPSC-CMs were incorporated into the 3D EHT model to study their functional properties at the tissue level. First, the model was optimized with a long-term follow-up to define the best timepoints for our study. We cultured EHTs and assessed the tissue remodeling and functionality twice a week for ~3 months. As shown in Figure 5A,B, the EHT was remodeled over time, becoming thinner but stable, from week 1 until the end of the experiment. In parallel, beating frequency, force, and contraction and relaxation times were tracked (Figure 5C–F). Before the first week, the force on the EHTs was not strong enough to be quantified, even though contracting areas were observed microscopically in the tissue already 1 day after the EHT production. Contractile areas became larger and started connecting and synchronizing with other areas during the first week. Then, after the first week, all contractile parameters were quantified and evolved until most of them reached a plateau phase and became stable before day 28 (Figure 5C–F).

**Figure 6:**
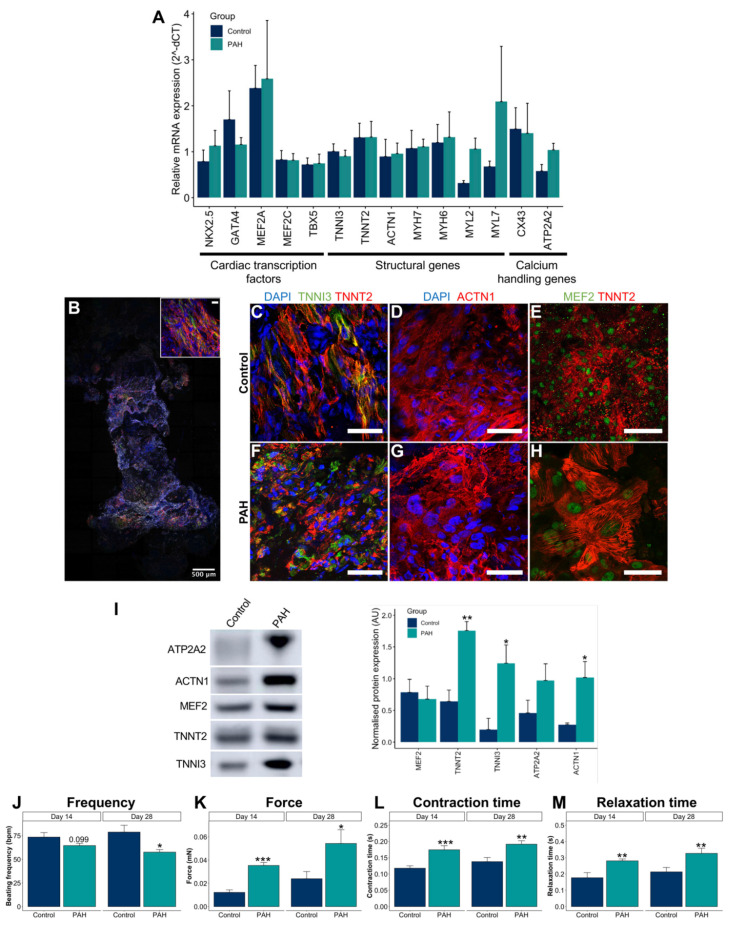
Control and PAH EHT characterization at the gene, protein, and functional levels. (**A**) Gene expression of cardiac transcription factors (NKX2.5, TBX5, MEF2A, MEF2C, and GATA4), structural genes (ACTN1, MYH6, MYH7, MYL2, MYL7, TNNI3, and TNNT2), and calcium-handling genes (ATP2A2 and CX43) genes for the control and PAH EHTs (mean + SD, N ≥ 7 control and N ≥ 7 PAH). (**B**) Representative whole-mount staining of a 3D EHT for TNNI3 (green), TNNT2 (red), and vimentin (grey). Scale bar = 500 µm (and 50 µm in the upper right magnification). (**C**–**H**) Representative images to confirm sarcomeric ACTN1 (red), TNNI3 (green), and TNNT2 (red) contractile markers, and MEF2 nuclear expression (green) on the control and PAH EHTs. Scale bars = 50 µm. (**I**) Representative Western blot bands and protein quantification of ACTN1, MEF2, TNNT2, TNNI3, and ATP2A2 cardiac markers on the control and PAH EHTs (mean + SD, N = 3 control, and N = 6 PAH). (**J**–**M**) Beating frequency (bpm), force (mN), contraction time (s), and relaxation time (s) in the control and PAH EHTs under spontaneous beating conditions at baseline (day 14) and at the end of the experiment (day 28) (mean + SD, N = 30 control, and N = 60 PAH). * *p* < 0.05, ** *p* < 0.01, *** *p* < 0.001.

Subsequently, the histology and expression of main cardiac markers of the EHTs were characterized. Both control and PAH EHTs expressed early and late cardiac markers, such as cardiac transcription factors, structural genes, and calcium-handling genes (Figure 6A). Histologically, iPSC-CMs aligned along the long axis of the EHT (Figure 6B) and expressed main markers, such as MEF2 in the nuclei and ACTN1, TNNI3, and TNNT2 in the sarcomeres (Figure 6C–H). The protein expression of MEF2, TNNT2, TNNI3, ATP2A2, and ACTN1 was also demonstrated and quantified for both control and PAH EHTs (Figure 6I). Interestingly, increased TNNT2, TNNI3, and ACTN1 were observed in PAH EHTs compared with in control EHTs.

Furthermore, spontaneous contractility of 3D EHTs was assessed through video optical recording on the EHT device at day 14 to study baseline characteristics as well as at day 28, which was the end of the experiment. PAH EHTs showed significantly increased force, contraction time, and relaxation time compared with control EHTs and a reduced beating frequency (Figure 6J–M).

Summing up, EHTs showed measurable forces from the first week, which remained stable after 1 month of EHT production, and expressed the main cardiac markers at the gene and protein levels. In addition, PAH EHTs have a much greater force compared with control EHTs, along with a prolonged contraction.

### 3.5. Increased Afterload on Control and PAH EHTs 

**Figure 7:**
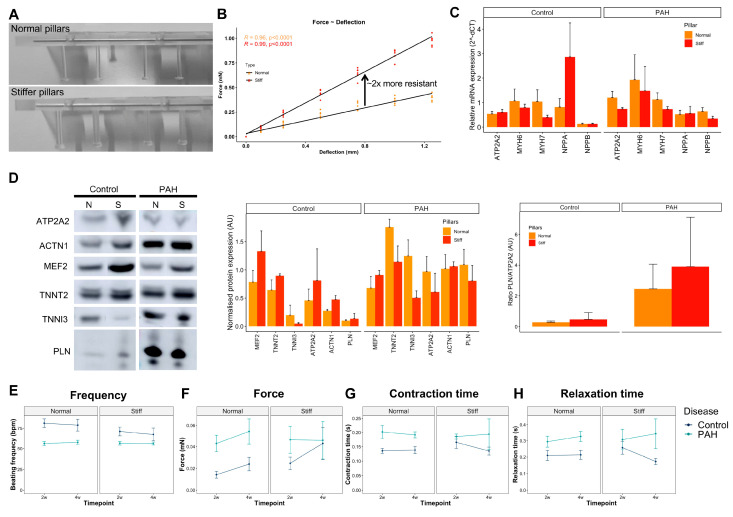
Increased afterload on control and PAH EHTs. (**A**) Image of normal and stiffer silicone posts. (**B**) Elastic characterization of normal and stiffer silicone posts plotting the force measured at different post deflections to calculate their resistance and spring constant (mN/mm). (**C**) Gene expression of structural and hypertrophic markers (ATP2A2, MYH6, MYH7, NPPA, and NPPB) on the control and PAH EHTs cultured with normal or stiffer silicone posts for 28 days (mean + SD, N ≥ 9 normal and ≥11 stiff control EHTs, and N ≥ 17 normal and ≥11 stiff PAH EHTs). (**D**) Representative Western blot bands and protein quantification of ACTN1, MEF2, TNNT2, TNNI3, PLN, and ATP2A2 cardiac markers on the control and PAH EHTs cultured with normal or stiffer silicone posts for 28 days (mean + SD, N = 3 normal and 2 stiff control EHTs, and N = 6 normal and 3 stiff PAH EHTs). (**E**–**H**) Beating frequency (bpm), force (mN), contraction time (s), and relaxation time (s) in the control and PAH EHTs cultured with normal or stiffer silicone posts under spontaneous beating conditions at 2 and 4 weeks (mean ± SD; N = 23 normal and 17 stiff control EHTs, and N = 17 normal and 8 stiff PAH EHTs).

At baseline conditions, control and PAH EHTs had different contractile features, but still did not fully recapitulate the typical failing phenotype of PAH right heart dysfunction, such as cardiac hypertrophy. Hence, we decided to mimic the increased afterload of the PAH right ventricle with a mechanical approach using stiffer posts to produce the EHTs. We custom-made and characterized silicone posts with higher stiffness. The stiffer silicone posts were thicker and showed twofold more resistance to deflection compared with normal silicone posts (Figure 7A,B).

Afterwards, the control and PAH EHTs were produced using normal and stiffer silicone posts and maintained for 28 days at 37 °C in a 7% CO_2_-humidified cell culture incubator. Unfortunately, several EHTs did not sustain the higher resistance for 28 days that well and detached from the posts or became thinner and broke before the end of the experiment, reducing the final number of EHTs.

We also investigated the gene and protein expression of several hypertrophic markers on the EHTs cultured for at least 14 days on stiffer posts, and no differences were observed between normal and stiffer posts on both control and PAH EHTs (Figure 7C,D), except trends for reduced ATP2A2 gene expression (*p* = 0.078), reduced NPPB gene expression (*p* = 0.065), and reduced MYH7 gene expression (*p* = 0.079) on stiffer posts compared with normal posts in PAH EHTs. Moreover, no differences between PLN/ATP2A2 ratios between control and PAH EHTs were present.

EHT functionality was also tracked over time (2 and 4 weeks), and despite clear deviations between force, and contraction and relaxation times on stiffer posts for the control and PAH EHTs, no statistically significant differences were observed because of the high variability and the low numbers at the end of the experiment, as only EHTs that reached 4 weeks were considered in the analyses (Figure 7E–H).

Taken together, an explicit hypertrophic response was not observed neither in the control nor in PAH EHTs after culturing them in stiffer posts.

## 4. Discussion

In this study, we successfully obtained iPSCs from a female PAH patient and a healthy control subject and differentiated these cells into iPSC-CMs. Moreover, we demonstrated intrinsic contractile differences between the control and PAH iPSC-CMs at both the 2D and 3D levels. Unfortunately, the increased afterload strategy tested on the 3D EHTs did not reveal a clear hypertrophic phenotype at the gene, protein and functional levels. To discuss the findings we found on our PAH models as well as some of the features of the 2D and 3D models, we first explore current knowledge on 2D and 3D cardiac models, and their (dis)advantages and put our findings into context with the clinical data available for PAH.

### 4.1. 2D Cardiac Models and Findings

First, in vitro studies were performed with primary human cardiomyocytes derived from patients. These samples were collected during invasive procedures, mostly from right atrial origin, and had a limited lifespan of ~2 days [18]. Later, iPSCs technology allowed for the development of an indefinite source of patient-specific stem cells carrying the patient’s genetic background that can be differentiated into any specific cell type of the human body, such as cardiomyocytes, which helped to study disease pathomechanisms. In fact, most cardiovascular diseases with a genetic cause have been studied in human iPSC-CMs and confirmed some phenotypic abnormalities that were previously described in native patients’ cardiomyocytes [19]. Although iPSC-CMs exhibit large phenotypic heterogeneity because of their immaturity, the resulting iPSC-CM population allows for the study of different parameters, such as morphology, calcium handling, and contractility. These 2D in vitro models have clear advantages, as they are well established, comparable with abundant previous literature, cost-effective, and easy to study under a bright field microscope and allow for high throughput analyses when working at the single cell level or even with small beating areas, as we achieved by using the CytoCypher Multicell High Throughput System (CytoCypher BV).

The wide variety of iPSCs cardiac differentiation protocols mimic the embryonic development of the heart, from mesoderm induction to cardiomyocyte specification, by subsequent media changes applied to a monolayer of cells or floating embryoid bodies [7,20]. Interestingly, our PAH iPSCs successfully differentiated towards iPSC-CMs despite carrying a heterozygous mutation in the BMPR2 gene, to which bone morphogenetic protein 4 (BMP4) preferentially binds [21]. Indeed, BMP4 is widely used in the mesoderm induction of most cardiomyogenic differentiation protocols available in the literature as well as in our optimized protocol.

The same protocol was used and carried in parallel on our control and PAH iPSCs in order to obtain the iPSC-CMs; however, the PAH genetic background unraveled some inherent contractile features different from the healthy cells that need further research.

### 4.2. 3D Cardiac Models and Findings

Tissue engineering results from the combination of biology and engineering with the aim to recapitulate the extremely complex human physiology. Cardiac tissue engineering integrates different matrices or scaffolds with various cell types to mimic the multicellular cardiac tissue [22]. In fact, iPSCs provided limitless quantities of well-characterized healthy or disease-specific cardiac and non-cardiac cells that enabled the generation and scale-up of human (patho)physiological engineered cardiac tissues.

Cardiac tissue engineering has been used for disease modelling and drug screening, hereby progressing into the field of precision medicine, and has numerous advantages over 2D iPSC-CMs cultures [23]. First, 3D models are a more accurate representation of the human physiology and, therefore, more predictive than cells seeded on top of coated plastic surfaces. Second, iPSC-CMs were reported to be more mature in a 3D environment than a 2D monolayer [24,25]. Third, 3D models can be multicellular, thereby allowing for the interaction between different cell types and the extracellular matrix and, consequently, evidencing a higher degree of structural complexity [23]. Moreover, microfluidics or vascularization can also be incorporated into these 3D models [14]. Last but not least, 3D models help to reduce the use of animal models in clinical translation. In this line, animal models failed to replicate human symptomatology and, especially in PAH, where there is no animal model that completely shows both the pulmonary and cardiac features.

Our 3D EHTs showed lower force compared with published literature with commercial or control human iPSC-CMs [15], probably because of the source of iPSCs and the cardiac differentiation protocol used. Nevertheless, as the same maintenance and differentiation protocols were used for both control and PAH iPSCs, discrepancies between them are of interest and confirmed, once more, the inherent contractility differences observed at 2D.

### 4.3. Disease Phenotype

Our work is a first step towards a more accurate model to study PAH-induced RHD that confirms the relevance of using patient-derived cells and the inherent contractile disparities between both populations at 2D and 3D levels. One limitation of this study is that we used iPSCs clones obtained from only one healthy subject and one PAH patient because working with more clones from other subjects and patients would be too labor-intensive and difficult to carry out. Nevertheless, we used three iPSCs clones per subject, performed different cardiac differentiation batches, and produced several EHTs per clone as a proof-of-principle. We were able to demonstrate functional differences both in 2D and 3D, which reflects previous findings of RV dysfunction in PAH patients, such as hypercontractility [26,27], and prolonged contraction and relaxation time [28,29]. These are the effects of the increased contractile proteins (i.e., ACTN1, TNNI3, and TNNT2) observed in our 3D PAH EHTs, a consequence of impaired BMP signaling because of a BMPR2 mutation [5], or a result of preserved epigenetic/environmental information that is maintained in the iPSCs [30], which should be investigated in more detail in future studies. Unfortunately, we were not able to induce hypertrophy in the 3D EHTs that were exposed to increased pressure overload. Nevertheless, we did show a distinctive response to pressure overload in control and PAH-derived EHTs where PAH-derived EHTs were not able to increase NPPA expression in contrast with control EHTs. In addition, the absence of hypertrophy in our 3D EHTs may be explained by the observation in experimental models that fibroblasts and macrophages are essential in the induction of hypertrophy in response to pressure overload [31,32]. Future experiments with multicellular EHTs will provide further insights in the interplay between different cell types (cardiomyocytes, fibroblasts, immune cells, and vascular cells) regulating the response to pressure overload [33].

Some limitations need to be overcome, and deeper investigations are needed. On one hand, the high-throughput analyses of the 2D iPSC-CMs led to more robust results despite using several cardiac differentiation batches. On the other hand, high variability and low number of specimens on EHT experimentation required multiple replicates and experiments to achieve representative results to confirm previous observations and to confirm higher complexity of the 3D model compared with the 2D model. In addition, after procedure optimization, new challenges and unexpected troubleshooting still appeared, such as sudden rupture of EHTs.

Finally, further research is needed to elucidate the pathomechanisms behind the different contractile performance of control and PAH models. Furthermore, more features of the dysfunctional right ventricle should be reproduced in the 3D model, such as hypertrophy or capillary rarefaction, to unravel the fate of the right ventricle. Consequently, multicellular models as well as environmental strategies (i.e., mechanical load, chronic pacing, hormonal treatment, and hypoxia) should be implemented and the resulting models need to be studied at the gene, protein, and functional levels in detail.

## 5. Conclusions

To conclude, we successfully generated iPSCs and iPSC-CMs from healthy subjects and PAH patients. We studied the baseline characteristics of 2D iPSC-CMs and found some functional differences, which were also confirmed in the 3D EHTs. This is the first version of a 3D model to study PAH-induced RHD. Further optimization will be performed and will consist of a multicellular model with a clear hypertrophic phenotype to represent a more complex 3D model to study PAH-induced RHD.

## Supplementary Materials

The following are available online at https://www.mdpi.com/article/10.3390/cells10123595/s1, Table S1: List of primer sequences used for the gene expression analysis. Movie S1: PAH iPSC-CM spontaneously beating at day 9.
