# Supplementary material for: Development of a 3-Dimensional Model to Study Right Heart Dysfunction in Pulmonary Arterial Hypertension: First Observations
Source: Cells. 2021 Dec 20;10(12):3595. doi: 10.3390/cells10123595 (PMC8700676; doi:10.3390/cells10123595)
Supplement: Supplementary file 1 [file cells-10-03595-s001.zip › Supplementary Table 1_ALVrev.pdf]

Supplementary Table S1. List of primer sequences used for the gene expression analysis

**Gene**	**Primer sequences**
Glyceraldehyde-3-phosphate dehydrogenase (*GAPDH*)	FWD: GGTCTCCTCTGACTTCAACA<br>REV: AGCCAAATTCGTTGTCATAC
Ribosomal protein L27 (*RPL27*)	FWD: TACAGCAGTGGAGGGAGACC<br>REV: TCTGAAGACATCCTTATTGACG
Hypoxanthine phosphoribosyltransferase 1 (*HPRT1*)	FWD: CCTGGCGTCGTGATTAGTGAT<br>REV: AGACGTTCAGTCCTGTCCATAA
NK2 homeobox 5 (*NKX2.5*)	FWD: CCAAGGACCCTAGAGCCGAA<br>REV: ATAGGCGGGGTAGGCGTTAT
T-box transcription factor 5 (*TBX5*)	FWD: CTGTGGCTAAAATTCCACGAAGT<br>REV: GTGATCGTCGGCAGGTACAAT
Myocyte enhancer factor 2A (*MEF2A*)	FWD: GGTCTGCCACCTCAGAACTTT<br>REV: CCCTGGGTTAGTGTAGGACAA
Myocyte enhancer factor 2C (*MEF2C*)	FWD: CCAACTTCGAGATGCCAGTCT<br>REV: GTCGATGTGTTACACCAGGAG
GATA binding protein 4 (*GATA4*)	FWD: GTGTCCCAGACGTTCTCAGTC<br>REV: GGGAGACGCATAGCCTTGT
Actinin alpha 1 (*ACTN1*)	FWD: CCACCCTCTCGGAGATCAAG<br>REV: TCCCTTCGCTTCTGAGTTAGG

Myosin heavy chain 6 (*MYH6*)	FWD: GCCCTTTGACATTCGCACTG<br>REV: CGGGACAAAATCTTGGCTTTGA
Myosin heavy chain 7 (*MYH7*)	FWD: ACTGCCGAGACCGAGTATG<br>REV: GCGATCCTTGAGGTTGTAGAGC
Myosin light chain 2 (*MYL2*)	FWD: TTGGGCGAGTGAACGTGAAAA<br>REV: CCGAACGTAATCAGCCTTCAG
Myosin light chain 7 (*MYL7*)	FWD: CAACGTGGTTCTTCCAACGTC<br>REV: CAACGTGGTTCTTCCAACGTC
Cardiac troponin I3 (*TNNI3*)	FWD: TTTGACCTTCGAGGCAAGTTT<br>REV: CCCGGTTTTCCTTCTCGGTG
Cardiac troponin T2 (*TNNT2*)	FWD: AATGGAGGAGTCCAAACCAAAG<br>REV: CCAAGTTGGGCATGAACGAC
ATPase sarcoplasmic/endoplasmic reticulum Ca2+ transporting 2 (*ATP2A2*)	FWD: ATGGGGCTCCAACGAGTTAC<br>REV: TTTCCTGCCATACACCCACAA
Gap junction protein, alpha 1 (GJA1) also known as Connexin43 (*CX43*)	FWD: GGGACAGCGGTTGAGTCAG<br>REV: TGTTACAACGAAAGGCAGACTG
Natriuretic peptide A (*NPPA*)	FWD: CAACGCAGACCTGATGGATTT<br>REV: AGCCCCCGCTTCTTCATTC
Natriuretic peptide B (*NPPB*)	FWD: GGGCGCTCCTGCTCCTGCTCTTC<br>REV: ACACCTGTGGGACGGGGGCTCTC
